# Patients with three or more primary melanomas: clinical-epidemiological study^☆^

**DOI:** 10.1016/j.abd.2022.12.003

**Published:** 2023-05-13

**Authors:** Tomas Fikrle, Barbora Divisova, Karel Pizinger

**Affiliations:** Department of Dermatovenereology, Charles University, Faculty of Medicine, University Hospital, Pilsen, Czech Republic

Dear Editor,

The incidence of malignant melanoma is growing worldwide and melanoma is responsible for most skin cancer-related deaths.^1^ Patients with melanoma undergo follow-up visits mainly to avoid disease progression.^2^ Moreover, the diagnosis of melanoma has been reported to be a risk factor for subsequent melanoma development.^3^ The majority of patients with multiple primary melanomas (MPM) have two primary tumors and only a small part of them has three or more primary melanomas (3PM). The literature is almost exclusively focused on the group of MPM patients as a whole and does not address patients with more than two primary tumors.^4^

We used a database of patients who followed up with malignant melanoma at the university department (Department of Dermatovenereology, University Hospital, Pilsen, Czech Republic) to find a cohort of patients with MPM. We focused on patients with 3PM to analyze them in detail in the range of retrospective studies.

We were interested in the gender, age at first melanoma excision, the total number of removed primary melanomas, the time interval between surgical excisions of MPMs, histopathological classification of all melanomas (AJCC classification at the time of tumor excision), location of all melanomas (head/neck, torso, upper limbs, lower limbs, other), development of nodal and/or distant metastases during follow-up. We also searched for melanoma in the family (to the second line of close relatives), coincidence with another type of cancer, phototype (I‒IV according to Fitzpatrick's classification), three or more episodes of sunburn in childhood (yes/no/I don't know), and the number and character of melanocytic nevi on the trunk and proximal parts of extremities (group 0 – no nevi, I ‒ up to 20 nevi, II – up to 50 nevi, III ‒ more than 50 common nevi, IV ‒ more than 50 nevi of different clinical appearance, including atypical nevi).

We identified 3641 patients who followed up for histopathologically confirmed malignant melanoma in the electronic database of our department, for whom all required information was available. We found 201 patients with MPM among them, which represents 5.52% of the cohort. Two primary melanomas were excised in 161 patients, and three or more primary melanomas (3PM) in the remaining 40 patients (1.10% of the whole cohort and 19.90% of the MPM group).

The 3PM group (40 patients) consists of 24 patients with three primary melanomas, 8 patients with four primary tumors, 4 patients with five primary tumors, 1 patient with six primary tumors, and 3 patients with seven primary melanomas (Figs. 1‒3).

**Figure 1:**
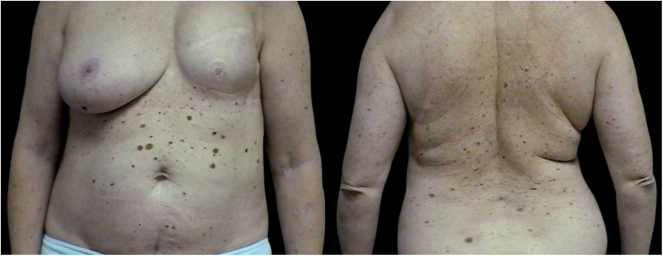
Clinical image. Female patient with seven primary melanomas excised between 2006 and 2020

**Figure 2:**
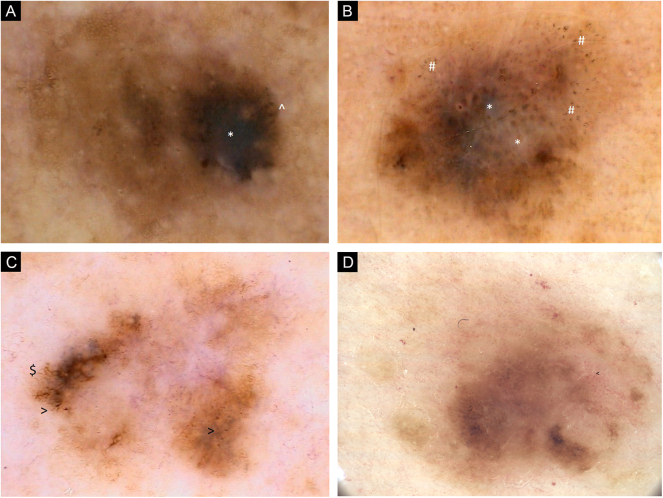
(A) Dermoscopic images of seven individual primary melanomas. 2006, 0.6 mm melanoma, upper extremity. Chaotic lesion, residual pigment network, eccentric hyperpigmented area with * Blue-gray veil and ^ Pigmented globules at the periphery. (B) Dermoscopic images of seven individual primary melanomas. 2008, 0.8 mm melanoma, upper extremity. Chaotic lesion, areas of regression with * Blue-white structures and # Peppering. (C) Dermoscopic images of seven individual primary melanomas. 2011, melanoma in situ, trunk. Chaotic lesion, central hypopigmentation, residual pigment network, $ Branched streaks and > Pigmented dots at the periphery. (D) Dermoscopic images of seven individual primary melanomas. 2017, melanoma 0.5 mm, trunk. Chaotic lesion, mainly structureless areas, multifocal areas of hyperpigmentation, < Dotted vessels

**Figure 3:**
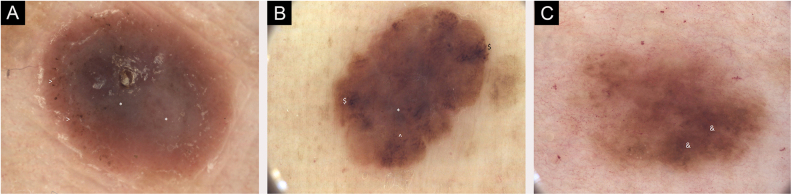
(A) Dermoscopic images of seven individual primary melanomas. 2018, melanoma 1.0 mm, lower extremity. Mainly structureless areas, central * Blue-white structures, peripheral > Pigmented dots, pink color. (B) Dermoscopic images of seven individual primary melanomas. 2019, melanoma 0.3 mm, lower extremity. Chaotic lesion, structureless areas, $ Branched streaks and ^ Pigmented globules at the periphery, central * Blue-gray veil. (C) Dermoscopic images of seven individual primary melanomas. 2020, melanoma 0.1 mm, trunk. Chaotic lesion, & Hyperpigmented areas with accented pigment network. Digital dermoscopic follow-up and total-body images were helpful in some decisions on thin melanoma excision

The group consists of 26 men (65.00%) and 14 women (35.00%) with a mean age of 57.48 years (range 24‒77 years) at the time of the first melanoma excision. The family history is positive for melanoma in 12 patients (30.00%). All phototypes I‒IV are represented and 35 patients (87.50%) were repeatedly sunburned in childhood. Patients with 3PM tend to have a high number of melanocytic nevi; 21 patients (52.50%; group IV) have multiple and clinically atypical melanocytic nevi.

We usually removed thinner subsequent primary melanomas in 3PM patients (1^st^ *melanoma* ‒ 22.50% in situ, 40.00% stage I, 37.50% stage II; 2^nd^ *melanoma* ‒ 57.50% in situ, 37.50% stage I, 5.00% stage II; 3^rd^ *melanoma* – 70.00% in situ, 27.50% stage I, 2.50% stage II). We excised only multiple in situ melanomas in 5 patients (three tumors in 3 patients and four tumors in 2 patients). Nineteen patients (47.50%) had excised only in situ or stage IA melanomas.

The first melanoma was most often located on the trunk (19 patients, 47.50%) and all primary melanomas occurred in the same location in 9 cases (22.50%).

The first and second melanomas in the same patient were frequently removed synchronously (0‒2 months interval; 10 patients, 25.00%) or within 2‒12 months (9 patients, 22.50%). On the contrary, the second melanoma occurred in more than 5 years in 14 patients (35.00%). The third melanoma was removed synchronously with the first tumor in 2 patients (5.00%), within 12 months, within 1‒5 years, and within 5‒10 years equally in 9 patients (22.50%) and in more than 10 years after the first tumor in the remaining 11 patients (27.50%). In 3 patients with seven primary melanomas, all seven tumors were removed within 11, 14, and 20 years, respectively. The youngest patient had a third primary melanoma excised at 35 years of age; in most patients (31 patients, 77.50%) this occurred after 60 years of age.

Nodal and/or distant metastases of melanoma occurred in 8 patients (20.00%) at 3PM. We found the coincidence of melanoma with another type of cancer in 14 patients (35.00%).

The internal morbidity of patients with 3PM did not significantly exceed the morbidity of the general population; mainly represented by arterial hypertension (52.50%), cardiac disease (30.00%), gall bladder/liver disease (17.50%), diabetes (15.00%), lipid metabolism disorders (15.00%), thyroid gland disease (10.00%).

Table 1 offers characteristics of 3PM group, as well as its comparison with the MPM group and single primary melanoma group ‒ SPM (control group of 1591 patients with SPM removed between January 2010 and December 2019). Both MPM and 3PM groups are more often represented by men, younger patients, lighter phototypes, patients with multiple and atypical melanocytic nevi, people with a history of sunburn, and a history of melanoma in the family compared to the SPM group. Moreover, patients with 3PM are more at risk of developing nodal and/or distant metastases of melanoma as well as another type of cancer. Differences in family history positivity, number and character of melanocytic nevi, and coincidence with skin cancer were found to be statistically highly significant when comparing 3PM vs. SPM, but also 3PM vs. MPM patients.

**Table 1:** Comparison of patients with single primary melanoma (SPM), multiple primary melanomas (MPM) and three or more primary melanomas (3PM)

	SPM	MPM	3PM
**Mean age at time of 1^st^ melanoma excision**^a^	60.28 years	58.48 years	57.48 years
**Number of patients**	**1591 (100%)**	**201 (100%)**	**40 (100%)**
**Gender**^b^			
Male	806 (50.66%)	117 (58.21%)	26 (65.00%)
Female	785 (49.33%)	84 (41.79%)	14 (35.00%)
**Family history positive**^c^	82 (5.15%)	25 (12.44%)	12 (30.00%)
**Phototype (Fitzpatrick's type)**^d^			
Type I	157 (9.87%)	24 (11.94%)	9 (22.50%)
Type II	612 (38.47%)	94 (46.77%)	13 (32.50%)
Type III	613 (38.53%)	62 (30.84%)	12 (30.00%)
Type IV	209 (13.13%)	21 (10.45%)	6 (15.00%)
**Repeated sunburn in childhood**			
Yes	1223 (76.87%)	168 (83.58%)	35 (87.50%)
No	296 (18.60%)	20 (9.95%)	5 (12.5%)
I don't know	72 (4.53%)	13 (6.47%)	0 (0.00%)
**Number/type of melanocytic nevi**^e^**(on the trunk and proximal extremities)**			
Group 0 (no nevi)	195 (12.26%)	8 (3.98%)	0 (0.00%)
Group I (up to 20 common nevi)	838 (52.67%)	70 (34.82%)	7 (17.50%)
Group II (up to 50 common nevi)	334 (20.99%)	52 (25.87%)	5 (12.50%)
Group III (more than 50 common nevi)	154 (9.68%)	34 (16.92%)	7 (17.50%)
Group IV (more than 50 nevi, atypical nevi)	70 (4.40%)	37 (18.41%)	21 (52.50%)
**Histopathologic classification of 1^st^ primary melanoma (AJCC)**^f^			
Stage IA	435 (27.34%)	40 (19.90%)	9 (22.50%)
Stage IB	599 (37.65%)	74 (36.82%)	14 (35.00%)
Stage IIA	205 (12.88%)	31 (15.42%)	2 (5.00%)
Stage IIB	128 (8.05%)	21 (10.45%)	5 (12.50%)
Stage IIC	136 (8.55%)	27 (13.43%)	8 (20.00%)
In situ	88 (5.53%)	8 (3.98%)	2 (5.00%)
**Location of 1^st^ primary melanoma**			
Trunk	788 (49.53%)	107 (53.23%)	19 (47.50%)
Upper limbs	329 (20.68%)	39 (19.40%)	9 (22.50%)
Lower limbs	271 (17.03%)	30 (14.93%)	8 (20.00%)
Head/neck	196 (12.32%)	25 (12.44%)	4 (10.00%)
Other	7 (0.44%)	0 (0.00%)	0 (0.00%)
**Coincidence with another type of cancer**^g^	382 (24.01%)	41 (20.40%)	14 (35.00%)
**Non-melanoma skin cancer**^h^	169 (10.62%)	26 (12.94%)	11 (27.50%)
**Nodal and/or distal metastases of melanoma**^i^	173 (10.87%)	25 (12.44%)	8 (20.00%)

SPM, Patients with single primary melanoma; MPM, patients with multiple (2 or more) primary melanomas; 3PM, Patients with 3 or more Primary Melanomas.

Statistical comparison of results for groups 3PM vs. SPM, 3PM vs. MPM, 3PM vs. SPM + MPM. Statistical data analysis was performed using SAS software (SAS Institute Inc., Cary, NC, USA). Non-parametric tests (Wilcoxon test, Median test) were used to compare the distributions of the investigated parameters between the tested groups. Differences in frequencies were tested using Fisher's exact test or Chi-Square test. Statistical significance was set at 5% (p < 0.05). Statistically significant differences are highlighted in bold and relevant parameters with*.

a Mean age at time of 1st melanoma excision: 3PM vs. SPM (p = 0.2021), 3PM vs. MPM (p = 0.5911), 3PM vs. SPM + MPM (p = 0.2303).

b Gender: 3PM vs. SPM (p = 0.0504), 3PM vs. MPM (p = 0.4246), 3PM vs. SPM + MPM (p = 0.0665).

c Family history positive: 3PM vs. SPM (p < 0.0001), 3PM vs. MPM (p = 0.0049), 3PM vs. SPM + MPM (p < 0.0001).

d Phototype: 3PM vs. SPM (p = 0.0623), 3PM vs. MPM (p = 0.1815), 3PM vs. SPM + MPM (p = 0.0709).

e Number/type of melanocytic nevi: 3PM vs. SPM (p < 0.0001), 3PM vs. MPM (p = 0.0001), 3PM vs. SPM + MPM (p < 0.0001).

f Histopathologic classification of 1^st^ primary melanoma (AJCC): 3PM vs. SPM (p = 0.3644), 3PM vs. MPM (p = 0.6300), 3PM vs. SPM + MPM (p = 0.4564).

g Coincidence with another type of cancer: 3PM vs. SPM (p = 0.1094), 3PM vs. MPM (p = 0.0445), 3PM vs. SPM + MPM (p = 0.0944).

h Coincidence with non-melanoma skin cancer: 3PM vs. SPM (p = 0.0028), 3PM vs. MPM (p = 0.0196), 3PM vs. SPM + MPM (p = 0.0010).

i Nodal and/or distal metastases of melanoma: 3PM vs. SPM (p = 0.0695), 3PM vs. MPM (p = 0.2039), 3PM vs. SPM + MPM (p = 0.0763).

In conclusion, we can state that MPM patients have more expressed well-known risk factors for malignant melanoma, which is even more pronounced in 3PM patients.^5^, ^6^ According to our results, 3PM patients also have an increased risk of melanoma metastases and coincidence with another type of cancer.^7^, ^8^ Long-term follow-up of patients with melanoma, as well as patients with melanoma risk factors, is important.^9^, ^10^ The genetic testing of MPM patients and their tumors would be interesting and could be the subject of further research.

## Financial support

None declared.

## Author’s contributions

Tomas Fikrle: Approval of the final version of the manuscript; critical literature review; data collection, analysis and interpretation; effective participation in research orientation; intellectual participation in propaedeutic and/or therapeutic; management of studied cases; preparation and writing of the manuscript; statistical analysis; study conception and planning.

Barbora Divisova: Critical literature review; data collection, analysis, and interpretation; intellectual participation in propaedeutic and/or therapeutic; management of studied cases.

Karel Pizinger: Approval of the final version of the manuscript; effective participation in research orientation; manuscript critical review.

## Conflicts of interest

None declared.
